# Supplementary material for: Content-rich biological network constructed by mining PubMed abstracts
Source: BMC Bioinformatics. 2004 Oct 8;5:147. doi: 10.1186/1471-2105-5-147 (PMC528731; doi:10.1186/1471-2105-5-147)
Supplement: Additional File 5 — The original Chilibot query results of the term "long-term potentiation (LTP)" and 22 other terms, limiting the latest references analyzed to the years 1990, 1995, 2000, and 2004. [file 1471-2105-5-147-S5.bz2 › chilibotAdditionalFile5/ltp1995/html/TAU_ATF.html]

 


 **TAU** and **ATF** 
  
Found 2 abstracts in PubMed,  **2 abstracts were retrieved and analyzed**.  


---

 Search Google  |
 PDF files only 
|  EDU domain only 

---

- J Biol Chem, 1993   **Temporal and tissue specific interactions involving novel transcription factors and the proximal promoter of the mouse Pdha 2 gene.** .
  We have determined by deletion analysis that the most proximal region of the Pdha 2 promoter between nucleotide position 187 to 22 harbors a transcriptionally active core.
  This core promoter directs high levels of CAT chloramphenicol acetyltransferase reporter gene transcription in HeLa cells.
  DNase I footprinting of the proximal promoter revealed four regions of protection.
  One of these contains the consensus sequence for the Sp1 binding site and another the **ATF** CREB binding site.
  The cis sequences of the remaining two protected regions designated MEP 2 and MEP 3.
  Mouse E1 alpha Promoter site show no apparent consensus homology with cis elements of other known transcription factors.
  Results of electrophoretic mobility shift assays confirm that the **ATF** CREB and MEP binding sites interact in a characteristic and specific manner with factors present in nuclei of both testis and somatic tissue.
  The factor which recognizes the MEP 3 motif appears to be ubiquitous.
  . . . the MEP 2 protein complexes were tissue specific.
  Interestingly, formation of a complex involving MEP 2 and a putative testis specific binding factor **tau** MEP 2BF is first observed in the testis of 2 week old mice, this correlates with the expression of Pdha 2.
  In contrast, the formation of complexes between the MEP 2 binding site and a somatic variant of MEP 2BF sigma MEP 2BF decreases in the testis as spermatogenesis proceeds.
  Our results suggest that 1 the MEP 2 binding factors are temporally regulated during spermatogenesis, and 2 interactions involving these factors with the MEP 2 cis element may be important for modulating Pdha 2 expression.

  - Neuron, 1993   **Transcriptional response to cAMP in brain specific distribution and induction of CREM antagonists.** .
    Changes in cAMP levels are often associated with the modulation of neuronal function.
    The CREM gene encodes both antagonists and activators of the cAMP dependent transcriptional response by alternative splicing.
    CREM transcripts in rat brain show a characteristic pattern of expression, being specific for the inner layer of the cerebral cortex, anterior thalamus, hippocampus, and hypothalamus.
    Strikingly, the CREM transcripts correspond to the antagonist isoforms in these areas, suggesting a down regulatory role for CREM in brain.
    in contrast, the expression of CREM **tau** and CREB activators is more diffuse and generalized.
    In the supraoptic nucleus, CREM expression is induced after osmotic stimulus.
    Importantly, this demonstrates physiological inducibility of CREM, which is novel within the CRE **ATF** family.
